# Effects of Chinese medical pricing reform on the structure of hospital revenue and healthcare expenditure in county hospital: an interrupted time series analysis

**DOI:** 10.1186/s12913-021-06388-2

**Published:** 2021-04-26

**Authors:** Mengling Liu, Mingyuan Jia, Qian Lin, Jiawei Zhu, Dong Wang

**Affiliations:** 1 School of Health Management, Southern Medical University, No.1023 Shatai Road, 510515 Guangzhou, China; 2 School of Economics and Management, South China Normal University, No. 55 Zhongshan Road, 510631 Guangzhou, China; 3 Nanfang Hospital, Southern Medical University, No.1838 Guangzhou North Road, 510515 Guangzhou, China; 4 Department of Occupational Health and Occupational Medicine, Guangdong Province Key Laboratory of Tropical Disease Research, School of Public Health, Southern Medical University, No.1023 Shatai Road, 510515 Guangzhou, China

**Keywords:** Zero Mark-up Drug policy, Zero Mark-up Medical Consumable policy, Medical pricing reform, Revenue structure, Healthcare expenditure per capita

## Abstract

**Background:**

China has initiated a medical pricing reform to combat the overuse of drugs and relieve the financial burden of patients. This paper aims to analyze the effect of medical pricing reform on revenue structure and healthcare expenditure of county public hospitals in Guangdong province.

**Methods:**

Based on the monthly data from January 2013 to August 2019, we use interrupted time series design to evaluate the effects of medical pricing reform on healthcare expenditure in both outpatients and inpatients. A counterfactual is also established to examine the net effect of the policy.

**Results:**

The proportion of drug revenue decreased from 35 % to 2015 to 29.7 % in 2019, and the revenue from medical services and inspection increased 3.2 and 3 % respectively. Meanwhile, the increasing trend of total expenditure and its main components is slowed down, especially the drug expense and medical consumable expense for inpatients after the Zero Mark-up Drug policy (coefficient = -18.76, *p* < 0.01; coefficient = -13.41, *p* < 0.01, respectively). However, the growth of inspection expense for outpatients continues to increase, while the healthcare expenditure for inpatients experiences an instant increase after the Zero Mark-up Medical Consumables policy. In terms of the net effect, most of healthcare expenditure in both outpatient and inpatient experienced a negative net growth from 2015 to 2019.

**Conclusions:**

The medical pricing reform is a valuable attempt in controlling the unreasonable increase of medical expenses. In the meantime, the unexpected increase in inspection expenditure and insufficient compensation from medical service adjustment should draw the attention of the policymakers.

## Background

Over the past decade, China has initiated the medical pricing reforms, including the Zero Markup for Drug policy (ZMD) in 2009 and Zero Markup for Consumables (ZMC) in 2017, which is the central component of the newest round of health system reform [1]. The medical pricing reforms removed all of the markups at the price of drug and medical consumables that were previously allowed in public hospitals, and adjust the medical price structurally to compensate the policies-related losses. The purpose of medical pricing reform is to cut off the economic linkage between hospitals revenue and drug and consumable sales, in order to change the behavioral incentives of medical providers and reduce the healthcare expenditure for patients. Meanwhile, it is also aimed to enhance the value of technical work performed by medical personnel to rectify the twisted pricing system.

Generally speaking, the implementation of medical pricing reforms has a specific historical background and realistic reasons. Since 1978, a series of policy interventions were implemented to promote the marketization in the health system [2, 3]. At that time, the proportion of government subsidy in public hospitals’ total revenue fell from 60 % to 1980 to 11 %in 2009, whereas the drug sales revenue rose rapidly to 42 % for the drug markup policy [4]. The Drug Markup policy could date back to the 1950 s when the public hospitals were allowed to add a 15 % markup on the actual purchase price of medicine [5, 6]. Additionally, public hospitals have widely implemented an incentive system since the mid-1990 s, which paid a lower baseline salary and awarded a high bonus directly related to hospitals’ total revenue [7, 8]. Motivated by the revenue-related incentive system and drug markup policy, Chinese physicians prescribed more high-priced drugs and overused unnecessary medicines to increase hospitals’ revenue as well as to maximize their own income [9].In other words, there was a financial incentive that encouraged physicians to induce the demand of patients in drugs and medical service and overprescribe medicines according to profit margin rather than cost-effectiveness and clinical efficacy [10].

As such, the medical expenses were growing rapidly, especially the drug expenses which accounted for over 50 % of the total medical expenditure per outpatient visit and over 40 % per inpatient admission in 2012 [11]. Moreover, it is estimated that half of the antibiotic prescriptions in China are medically unnecessary [12]. Overuse and misuse of antibiotics is not only an important reason for high medical expenditure but also has long-term negative health effects, like antimicrobial resistance. Such profit-seeking behaviors ran counter to healthcare providers’ professional ethics and norms that made the patients’ benefit the highest. Difficulty and high expenses of medical service became a severe social problem [13].

In view of this situation, China has initiated the first round of medical pricing reform in 2009 which was known as the Zero Markup for Drug policy (ZMD) [14]. According to the ZMD policy, public hospitals are required to remove all the profit margin that was previously allowed on the actual drug purchase price [15]. To ensure policy sustainability, the ZMD policy is also featured with an adjustment in medical price, including an increase in the price of medical services and a decrease in medical inspection fees [1]. The medical services involve the great experience, skill, and technical work of medical personnel, like acupuncture, surgical operations, and nursing care, etc. By increasing the medical services price, one of the policy’s objectives is to change the behavioral incentives of medical providers indirectly from drug selling to high-quality medical service providing.

Although the first round of medical pricing reform has made some progress, the profit-seeking behavior of public hospitals formed over decades cannot be sufficiently reversed overnight. Some researchers have found that compensation from government subsidies and medical pricing adjustment is insufficient to offset the shortfall caused by ZMD policy [16, 17]; and public hospitals may seek other income sources to make up for the significant reduction in drug sales income, such as high-value medical consumables and medical inspections [17, 18]. Medical consumables can be divided into low value and high value. Low-value medical consumables are those basic and widely-used consumables, referring to disposable syringe needles, hemostatic gauze, infusion apparatus, etc. And high-value medical consumables refer to the sanitary materials with high price or that need to be implanted or intervened in the human body, such as heart stents, artificial joint, angiography catheter, etc. Actually, public hospitals are required to control the proportion of drug income and medical consumables within 30 and 20 % respectively before 2017. In addition, 5–10 % markup was still allowed for selling medical consumables after 2009, which drove the inappropriate use of consumables by similar financial incentives in drug markup policy. It means that medical consumables account for a large proportion of the hospitals’ revenue, and it may become a new driver of healthcare costs escalation [19, 20].

In this context, China proposed the latest round of medical pricing reform in 2017, known as the Zero Markup for Consumables policy (ZMC), to further reform the healthcare pricing system. Similarly, public hospitals were required to remove all of the profit margins on medical consumables and carry out a structural adjustment in medical services price to offset the reduction of medical consumable revenue. Recently, the ZMC policy was implemented firstly in some provinces, like Fujian, Anhui, and Guangdong, and being rolled out gradually in every public hospital throughout the country.

There have been some researches conducted to evaluate the effects of the ZMD policy implemented on different hospitals in different regions in China. For example, Zhou et al. [21] analyzed the impact of ZMD policy on county hospitals in Shaanxi through the difference-in-difference (DID) method and found a reduction in drug expenditure and total expenditure in both outpatients and inpatients. Zeng et al. [20] had founded an unexpected and rapid growth in medical consumables expenditure. As the existing researches showed, the ZMD policy had reduced the drug cost and healthcare expenditure in the short term whereas its effects on controlling the growth of medical expenditure were questionable with some unintended consequences.

Although the existing literature yielded major advances on the preliminary impacts of ZMD policy, most of the researches on ZMD policy used the short-term data pre and post-reform [19–26]. It’s difficult to conclude the trend change in the future, and it’s uncertain whether the policy impacts return to the previous trend after a short period. Due to the short-term analysis, the unexpected policy impacts and substitution effects observed by researchers are also different. Therefore, it’s necessary to review the ZMD policy at this critical point, analyze the long-term effects and provide empirical evidence to prove the unintended consequences. Moreover, there is little research to examine the effects of the newly issued ZMC policy on health expenses and hospital revenue structure.

Public county hospitals are the leader in the rural healthcare network, the main provider of healthcare in the Chinese healthcare system, and also the focus of medical reform in China. According to statistics, at least 85 % of patients choose the medical institution within county areas in 2018, especially county hospitals. Taking county hospitals as examples, our paper presents a new analysis on the effects of ZMD policy with 4 years of follow-up data and the preliminary effect of ZMC policy. We investigated how the ZMD policy and ZMC policy changed the structure of hospital revenue and healthcare expenditure, aiming to detect the positive influences and unintended consequences of the medical pricing reform. A counterfactual is also constructed to exclude the impact of natural growth trends and assess the net effect of medical pricing reform. The empirical fundamentals and evidence-based experience provided in this paper may help inform the further reform and shed light on other countries with similar systems.

## Methods

### Study setting

We conducted this study in Guangdong province, which is located in south China and acts as the pioneer of healthcare system reform in China. In response to the national policy, Guangdong province singled out some county hospitals as pilot units to conduct the ZMD policy in November 2012, and fully implemented it in county hospitals across the province in August 2015. According to the ZMD policy in Guangdong province, public hospitals are required to remove the 15 % mark-up for drugs with an “811” compensation measure. “811” compensation measure means that 80 % of the losses caused by ZMD policy is compensated by adjusting the price of medical services, 10 % by increasing the government funding, and the rest 10 % by the hospital itself. Besides, the ZMC policy in Guangdong province was launched and fully implemented at the end of 2018, when the ZMD policy’s reform costs were basically compensated. Unlike the ZMD policy, 100 % of the losses from the ZMC policy is compensated by further adjusting medical service price.

### Data sources and outcome indicators

According to Guangdong province’s geographical divisions, it can be divided into 4 regions of East Guangdong, West Guangdong, North Guangdong, and Pearl River Delta. Thus, we adopted the purposive sampling method to investigate eight county-hospitals from four regions respectively, which also represent the level of economic development in Guangdong province. Given the lag in policy implementation, we choose the time when the policy was fully implemented as the intervention point. Therefore, August 2015 and December 2018 are the intervention measure of ZMD policy and the intervention measure of ZMC policy, respectively.

Monthly data from January 2013 to August 2019 were collected, which covers the total number of outpatients, number of inpatients, and total service revenue in both outpatients and inpatients. The total service revenue also includes drug revenue, consumable revenue, inspection revenue (assay revenue), medical service revenue, and others. We define medical service revenue as the income from providing technical services, such as treatment, surgery, and nursing. These services involve the rich experience, high skill, and heavy labor of medical personnel. All of the data are converted to real terms based on the Consumer Price Index for Medical Services in Guangdong province.

Given the objective of medical pricing reforms, indicators on hospital revenue and healthcare expenditure in both outpatients and inpatients are designed and calculated. The indicators are the proportion of the major component revenue in total revenue, total expenditure per capita for outpatient and inpatient, and its major components, such as the drug expense per capita for outpatient. In this study, the sample ranges from 2013 to 2019, with 80 observations in total.

### Statistical analysis

An interrupted time series (ITS) design with two interventions is applied in this study to evaluate the effects of medical pricing reform. As the strongest quasi-experimental study, the ITS design can exclude the impact of typical and fairly constant confounding variables for evaluating the short-term effect and long-term effect of the intervention [27, 28]. We also construct a counterfactual without medical pricing reform and predict the pseudo value of indexes in the post-reform period, in order to evaluate the “net effect” of medical pricing reform [29, 30]. Usually, the ITS regression model with two interventions takes the following form:

$${\mathrm Y}_{\mathrm t}={\mathrm\beta}_0+{\mathrm\beta}_1{\mathrm T}_{\mathrm t}+{\mathrm\beta}_2{\mathrm P}_{1\mathrm t}+{\mathrm\beta}_3{\mathrm D}_{1\mathrm t}+{\mathrm\beta}_4{\mathrm P}_{2\mathrm t}+{\mathrm\beta}_5{\mathrm D}_{2\mathrm t}+{\mathrm\varepsilon}_{\mathrm t,}$$

Where Y_t_ denotes the healthcare expenditure in month t; T_t_ is the time since the start of the sample; P_1t_ is a dummy variable representing the first intervention (August 2015, preintervention periods 0, otherwise 1); D_1t_ is time after the first intervention; P_2t_ is a dummy variable representing the second intervention (December 2018, preintervention periods 0, otherwise 1); D_2t_ is time after the second intervention.

In our regression model, β_0_ represents the baseline level of the dependent variable; β_1_ is the slope until the introduction of the first intervention, representing the underlying pre-intervention trend; β_2_ (β_4_) measures the level change after the implementation of ZMD (ZMC) policy; β_3_ (β_5_) represents the difference of the slope between pre and post intervention. The Newey-West method was used to handle heteroscedasticity and autocorrelation.

## Results

### Structural change in hospitals’ service revenue

Table 1 shows the proportion change of major revenue from 2015 to 2019. Several findings are noteworthy. First, The proportion of drug revenue in total revenue dropped significantly by 5.4 % after the implementation of ZMD policy and continued to decrease by 1 % after ZMC policy in 2019. Second, the proportion of the revenue from medical consumables, inspection, and medical service increased and filled the space vacated by drug revenue percentage. It seems that the ZMC policy fails to fulfill its objective to decrease medical consumables income in the total revenue. In particular, the inspection revenue and medical service revenue increased by 3 and 3.2 % respectively. However, the inspection revenue became the main source of income in the county public hospitals, accounting for almost 1/3 of the total revenue.

**Table 1:** Proportion change of major revenue in total service revenue between pre and post of reforms (%)

	Pre of ZMD	Post of ZMD	Post of ZMC	Proportion Change		
	2015	2018	2019	2018/2015	2019/2018	2019/2015
Drug revenue	36.05	30.66	29.67	-5.39	-0.99	-6.38
Consumable revenue	10.50	10.92	11.50	0.42	0.58	1.00
Inspection revenue	29.23	32.47	32.20	3.24	-0.27	2.97
Medical service revenue	22.41	24.17	25.58	1.76	1.41	3.17
others	1.81	1.78	1.05	-0.03	-0.73	-0.76

### ITS analysis of the expenditure for outpatient

**Fig. 1:**
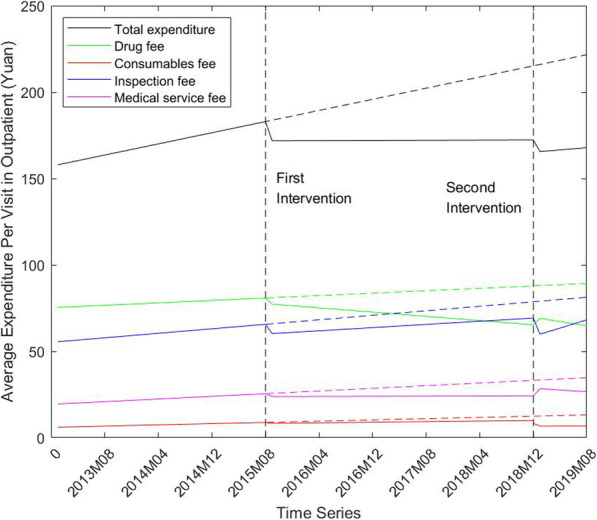
Change of average expenditure per capita in outpatient from 2013 to 2019

We use the ITS regression to analyze the change in level and trend on total expenditure, drug fee, medical consumable fee, inspection fee, and medical service fee. Figure 1 shows an intuitive reflection of the change in outpatient healthcare expenditure per capita with interventions. The ZMD policy (first intervention) resulted in a clear decline in total expenditure and its major components. However, the total expenditure, inspection fee, and consumable fee continued the decline since the ZMC policy, while the drug expense and medical service fee experienced a mild increase in 2019.

Table 2 reports the estimation result of our ITS regression for different dependent indicators. First, the ZMD policy has a negative effect on the total expenditure and its components. An instant decline can be found in all expenditure variables in outpatient with no statistical significance. In addition, the slope of all time trends experienced a slight decline and most of these changes are significant, such as the total expenditure (coefficient = -0.79, *p* < 0.05), drug fees (coefficient = -0.48, *p* < 0.01) etc. It means that the growth rates of most expenditure variables in outpatients decreased from month to month after ZMD policy.

**Table 2:** ITS regression results of the expenditure change in outpatient pre and post the ZMD policy and ZMC policy

	Total expenditure	Drug fees	Consumable fees	Inspection fees	Medical service fees
Intercept(β0)	157.15^**^	75.26^**^	6.04^**^	55.25^**^	19.34^**^
Baseline trend(β1)	0.81^**^	0.18	0.09^**^	0.33^*^	0.19^**^
Level change after ZMD(β2)	-11.06	-3.24	-0.58	-5.61	-1.65
Trend change after ZMD(β3)	-0.79^*^	-0.48^**^	-0.09^**^	-0.09	-0.18^**^
Level change after ZMC(β4)	-7.04^*^	4.34^**^	-1.64^*^	-10.57^**^	4.43^**^
Trend change after ZMC(β5)	0.31	-0.30	0.01	0.96^**^	-0.26

Two-tail *P* value: *p<0.05, **p<0.01. HAC (Newey-West) standard error

Second, the effects of the newly adopted ZMC policy on the expenditures are mixed. The level of total expenditure and consumable fee decreased statistically significantly (coefficient = -7.04, *p* < 0.05; coefficient = 1.64, *p* < 0.05, respectively). Additionally, the inspection fee per capita was reduced in the short term with a significant level change while it grows faster than before. (coefficient = -10.57, *p* < 0.01; coefficient = 0.96, *p* < 0.01, respectively). Different from inspection fee, the drug expense, as well as medical service, increased in level and decreased in the long run.

### ITS analysis of the expenditure for inpatient

**Fig. 2:**
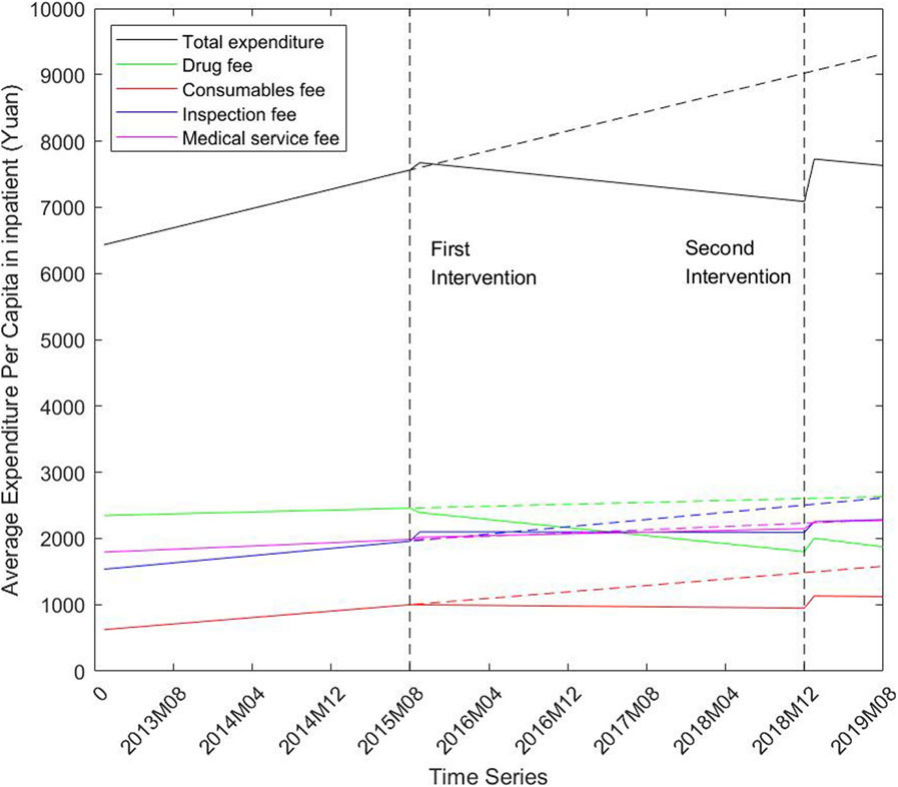
Change of average expenditure per capita in inpatient from 2013 to 2019

Figure 2 graphs the change in healthcare expenditure per capita and its major component expenses in inpatient. The graph illustrates a reduction in total expenditure and drug fees, and a rise in inspection fees under the intervention of medical pricing reform. Nevertheless, the total expenditure along with its components experienced a huge surge in the short term after ZMC policy, which is opposite to the trend change in the long run.

Table 3 shows the parameter estimates of our ITS regression for inpatients. The main findings are summarized as follows. First, the slopes of total expenditure, along with drug, consumables, and inspection expenditure, declined significantly after the ZMD policy implemented in 2015. Most of expenditure components experienced an instant level change with no statistical significance while a sharp rise was observed in inspection expense (coefficient = 142.12, *p* < 0.01). It means that the county hospitals were trying to increase the inspection volumes and items to compensate for the financial loss caused by ZMD policy. In addition, the ZMD policy has no effect on the medical service fee statistically.

**Table 3:** ITS regression results of the expenditure change in inpatient pre and post the ZMD policy and ZMC policy

	Total expenditure	Drug fees	Consumables Fees	Inspection Fees	Medical service fees
Intercept(β0)	6398.24^**^	2342.29^**^	608.60^**^	1520.53^**^	1786.90^**^
Baseline trend(β1)	36.43^**^	3.62	12.13^**^	13.66^**^	6.16^**^
Level change after ZMD(β2)	125.42	-52.01	0.55	142.12^**^	32.10
Trend change after ZMD(β3)	-51.48^**^	-18.76^**^	-13.41^**^	-13.82^**^	-2.87
Level change after ZMC(β4)	656.13^**^	221.17^**^	185.50^**^	157.31^*^	101.46
Trend change after ZMC(β5)	1.10	-3.53	-0.33	4.39	-0.57

Two-tail *P* value: *p<0.05, **p<0.01. HAC (Newey-West) standard error

Second, an instant and sharp level rise was observed in total expenditure and its components after the ZMC policy. In addition, most of the changes in the slope of time trend were not significant and meaningful. And the ZMC policy also has no effect on the medical service fee statistically. Both the obvious increase in level and the ineffective slope change are not in line with our expectation of the policy.

### The net effect of medical pricing reform

Using the pre-intervention level and trend estimates, we build a counterfactual prediction to evaluate the net effect of the medical pricing reform in this section. Table 4 shows that the medical pricing reform successfully fulfilled the policy objective to slow down the growth rate of healthcare expenditure, even cut the expenditure to a lower absolute level.

**Table 4:** The net effect of medical pricing reform on healthcare expenditure from 2015 to 2019

	Actual fee pre-	Actual fee post-	Forecasted fee post-	Actual growth rate	Forecasted growth rate	Net growth rate
Inpatient						
Total expenditure	7328.48	7680.83	9185.14	4.81%	25.33%	-20.53%
Drug fee	2414.35	1937.14	2618.87	-19.77%	8.47%	-28.24%
Consumables fee	929.28	1124.92	1536.90	21.05%	65.39%	-44.33%
Inspections fee	1916.45	2269.36	2565.23	18.41%	33.85%	-15.44%
Medical services fee	1907.78	2260.98	2257.87	18.51%	18.35 %	0.16%
Outpatient						
Total expenditure	173.53	166.73	218.81	-3.92%	26.09%	-30.01%
Drug fee	78.30	66.94	88.67	-14.51%	13.24%	-27.75%
Consumables fee	8.36	6.82	12.91	-18.42%	54.43%	-72.85%
Inspections fee	61.18	64.08	80.15	4.74%	31.01%	-26.27%
Medical services fee	23.96	27.52	34.10	14.86%	42.32%	-27.46%

The actual growth rates of total expenditure and most component expense are lower than the predicted value to varying degrees from 2015 to 2019, and the effects of the reform on expenditure for outpatient are larger than that on inpatient. Specifically, the actual growth rate of total expenditure per capita for outpatient showed negative growth in this period, which is 30.01 % lower than the predicted 26.09 %. Negative growth in real terms also could be observed in the drug expense, both in outpatient and inpatient. In addition, the medical pricing reform had its greatest impact on medical consumable expense, with − 44.33 % net growth rate for inpatient and − 72.85 % for outpatient.

The last thing to be noticed is the contrasting effects of the reform on medical service fees for outpatient and inpatient. It is shown that the medical pricing reform accelerated the growth of medical service fees for inpatient in slight degree, with 0.16 % net growth rate. However, the growth of medical service expense was slowed down, which was about 27.46 % lower than the counterfactual forecasted growth rate.

## Discussion

Our study contributes to the evidence for the impacts of medical pricing reform in China, especially for the unintended effects that cannot be revealed in short-term research. Specifically, the proportion of drug revenue in total revenue realized a considerable decline, and the increase of the medical service revenue compensated most of the loss in drug revenue. These changes are in line with the policy maker’s expectation to value medical service and cut the drug price, which has also been previously demonstrated [26, 31]. It means that medical pricing reform helps the public hospitals to alleviate their dependence on the inappropriate revenue from drug sales and motivate them to value technical work performed by medical personnel.

In addition, the growth rate of total medical expense for both outpatient and inpatient decreased considerably, as well as the growth rate of drug expense and medical consumable expense, which are directly related to medical pricing reform. Similar findings can also be found in previous studies [22, 23, 26]. Besides, the effects of the reform on medical expenses in outpatient are generally larger than that on inpatient, which is consistent with another study [32].

Not all the impacts of the medical pricing reform satisfy the policymakers, and there are also several unexpected effects on revenue structure and healthcare expenditure. One of the important policy goals is to increase the price of medical service and control the inspection fee. However, we find that the ZMD and ZMC policies have a limited effect on the medical service revenue. In particular, the negative net growth rate illustrates that the medical service expense has decreased over time, indicating that the compensation from adjusting medical service price is insufficient. We are not sure if this decreasing trend will continue or revert in the future. A possible reason is the slow adjustment of regulated prices in the complex health system, such as registration fee, surgery fee, and nursing fee [33]. Therefore, it is necessary to conduct continuous monitoring of policy impacts, adjust medical service price dynamically and perform corresponding interventions.

Additionally, a measurable increase in the proportion of inspection revenue in total revenue is found. In fact, the inspection revenue has already accounted for the highest proportion in 2019. Besides, the actual expense on inspection for inpatient grew even faster from 2015 to 2019 after the pricing reform. A similar sign of faster growth can also be found for outpatients after the ZMC policy when the slope of the time trend increases in our ITS regression. These results are consistent with the structural change in the revenue that inspection fees has become the main source of hospital revenue. The unexpected soaring of inspection fee indicates that the healthcare service provider tends to prescribe more examinations and increase the inspection service volumes to fill the deficit caused by the removal of drug markup [18].

We also find that the total expenditure per capita and its major component show an instant and considerable increase in inpatient around the time when the ZMC policy was implemented, which is an unexpected increase. In fact, we find a sizeable rise in expenditure about half years before the ZMC policy was formally started. The public hospital may increase the price of healthcare when the policy was still in the air but to be conducted soon. Furthermore, as the fee charged for inpatient is higher than the outpatient in China and the expense of outpatient is only partly insured by the public insurance, doctors were thus incented to admit outpatient as an inpatient [34]. Another important reason for the conspicuous increase in hospitalization expenditure is the improvement in medical service capacity and the ability to perform complicated surgery in county hospitals.

Although the medical pricing reform has made some positive progress, some unintended effects have weakened the whole effect. An unexpected increase in inspection expense indicated that medical pricing reform has caused the healthcare service provider to seek a new and potentially inappropriate income source. It means that a single pricing intervention seems to be unable to reverse the profit-driven behavior of public hospitals, and deal with the problem of supplier-induced demand. In order to rectify the improper behavior of health service providers in a true sense, it’s necessary to strengthen the role of government in the healthcare system to limit the excessive marketization of the medical industry. Meanwhile, the relevant policy should be promoted actively, such as the reform of the medical insurance system, the salary system in public hospitals.

After the implementation of medical pricing reform, the Diagnosis-intervention packet (DIP), a new medical insurance payment method, was launched as the linkage policy in Guangdong province. According to the DIP, hospitals’ revenue from the funds of medical security is related to treating diseases, treatment technology, treatment quality, and cost management [35]. In other words, the DIP may affect hospitalization expenditure by directly affecting the hospital management mode. How the DIP affects hospital management and hospitalization remains to be further studied for this reform is in the stage of exploration.

### Limitation

Although our study provides an empirical fundamental and evidence-based experience for the intended and unintended effects of ZMD policy and ZMC policy newly launched, our study has several limitations. First, we only conducted this study in some county public hospitals in Guangdong province and lacked a nationally representative sample, for the reason that the ZMC policy has not yet been implemented nationwide. Second, a control group should be helpful to separate the effects of medical pricing reform from other policy interventions that may occur during the medical pricing reform. Third, because hospitals implemented policy following area-specified schedule, the intervention points are slightly different for eight hospitals.

## Conclusions

Using the interrupted time series design to analyze the effects of medical pricing reform in China, our study showed that the ZMD and the ZMC policy is a valuable attempt in optimizing the revenue structure of hospitals and alleviating the financial burden on healthcare. The proportion of drug revenue decreased considerably. Medical service has increasingly become a more important source of hospital revenue in recent years. In addition, the rapid growth of healthcare expenditure, drug expense, and medical consumable expense has been slowed down effectively. It indicates that the medical pricing reform successfully restricted the profit-seeking behavior in drug and consumable sales and incented the healthcare providers to pay more attention to improving the service quality of medical personnel. However, the compensation from the adjustment of medical service price is still limited and the inspection expense increases unexpectedly, which may deteriorate the effect of ZMD and ZMC policies in the public hospital. The impact of the medical pricing reform needs to be further examined and monitored dynamically. And more research on ZMC policy is needed to assess the long effect on hospital’s revenue structure.

## Data Availability

The datasets generated during the current study are not publicly available in accordance with the project agreement, but are available from the corresponding author at dongw96@smu.edu.cn on reasonable request.

## Abbreviations

ZMD: The Zero Markup for Drug policy
ZMC: The Zero Markup for Consumables policy
ITS: An Interrupted Time Series
DIP: The Diagnosis-intervention Packet
